# Differential grey matter structure in women with premenstrual dysphoric disorder: evidence from brain morphometry and data-driven classification

**DOI:** 10.1038/s41398-022-02017-6

**Published:** 2022-06-15

**Authors:** Manon Dubol, Louise Stiernman, Johan Wikström, Rupert Lanzenberger, C. Neill Epperson, Inger Sundström-Poromaa, Marie Bixo, Erika Comasco

**Affiliations:** 1 Department of Women’s and Children’s Health, Science for Life Laboratory, Uppsala University, Uppsala, 753 09 Sweden; 2 Department of Clinical Sciences, Umeå University, Umeå, 901 85 Sweden; 3 Department of Surgical Sciences, Radiology, Uppsala University, Uppsala, 751 85 Sweden; 4 Department of Psychiatry and Psychotherapy, Medical University of Vienna, Vienna, 1090 Austria; 5 Department of Psychiatry, Department of Family Medicine, University of Colorado School of Medicine, Anschutz Medical Campus, Aurora, CO 80045 USA; 6 Department of Women’s and Children’s Health, Uppsala University, Uppsala, 753 09 Sweden

**Keywords:** Neuroscience, Depression

## Abstract

Premenstrual dysphoric disorder (PMDD) is a female-specific condition classified in the Diagnostic and Statical Manual—5th edition under depressive disorders. Alterations in grey matter volume, cortical thickness and folding metrics have been associated with a number of mood disorders, though little is known regarding brain morphological alterations in PMDD. Here, women with PMDD and healthy controls underwent magnetic resonance imaging (MRI) during the luteal phase of the menstrual cycle. Differences in grey matter structure between the groups were investigated by use of voxel- and surface-based morphometry. Machine learning and multivariate pattern analysis were performed to test whether MRI data could distinguish women with PMDD from healthy controls. Compared to controls, women with PMDD had smaller grey matter volume in ventral posterior cortices and the cerebellum (Cohen’s *d* = 0.45–0.76). Region-of-interest analyses further indicated smaller volume in the right amygdala and putamen of women with PMDD (Cohen’s *d* = 0.34–0.55). Likewise, thinner cortex was observed in women with PMDD compared to controls, particularly in the left hemisphere (Cohen’s *d* = 0.20–0.74). Classification analyses showed that women with PMDD can be distinguished from controls based on grey matter morphology, with an accuracy up to 74%. In line with the hypothesis of an impaired top-down inhibitory circuit involving limbic structures in PMDD, the present findings point to PMDD-specific grey matter anatomy in regions of corticolimbic networks. Furthermore, the results include widespread cortical and cerebellar regions, suggesting the involvement of distinct networks in PMDD pathophysiology.

## Introduction

Premenstrual dysphoric disorder (PMDD) is recognized in the DSM-5 as a hormone-related depressive disorder, specific to women’s mental health [1]. Women who suffer from PMDD experience affective, cognitive, and physical symptoms that peak during the late luteal phase of the menstrual cycle, and remit shortly after the onset of menses [1, 2]. Regrettably, they often spend many years struggling before being correctly diagnosed with PMDD and receiving appropriate care [3]. Variations in oestradiol and progesterone levels during the menstrual cycle are associated with the onset of PMDD symptoms [4], whereas low and stable levels of these hormones lead to symptom improvement [5]. The key mental symptoms of PMDD (affective lability, irritability, depressed mood and anxiety) point to anatomical and functional brain impairment, suggesting an impaired top-down inhibitory process involving limbic brain structures [6]. Albeit sparse, findings on the neural correlates of emotion processing, cognition, brain metabolism and neurotransmission in PMDD point to differences in the prefrontal cortex, insula, cerebellum, and amygdala [6], in comparison with healthy, naturally cycling women.

Notably, the neural underpinnings of PMDD remain poorly understood [7]. At present, only three studies have attempted to describe the neuroanatomical correlates of PMDD by use of voxel-based morphometry (VBM) or surface-based morphometry (SBM) [6]. According to these, women with PMDD might have greater cerebellar and left hippocampal cortex volumes and smaller parahippocampal cortex volumes compared to controls [8, 9], or they do not differ in brain structure from controls [10]. These three studies included small samples (*n* ≤ 20, on average *n* = 16), sometimes used asymptomatic phases or merged phases (i.e., combined the follicular and luteal phase) in their analyses, and did not account for important confounding factors such as age, total brain volume, and body mass index (BMI). Hence, a comprehensive examination of grey matter structure in PMDD compared to healthy controls, based on carefully designed analyses and larger samples of women ensuring higher statistical power, is still missing.

Smaller grey matter volumes (GMV) and thinner cortices have been observed in relation to several mental disorders sharing aspects of PMDD symptomatology, including anxiety disorders and major depression [11–14]. Further, individuals with major depression and anxiety disorders respectively display lower and greater gyrification of the cortex in comparison with healthy controls [12, 15]. On top of the disorder-specific brain structural alterations, there is increasing evidence that periods involving profound changes in ovarian hormone levels, like puberty, pregnancy and even the menstrual cycle, are associated with brain morphometric changes in women [7, 16, 17]. Therefore, we employed a combination of morphometric measures (i.e., GMV, cortical thickness, gyrification and sulcal depth) to yield a comprehensive analysis of grey matter-related brain structure in PMDD.

The present study aimed at investigating the grey matter structures that distinguish women with premenstrual dysphoric disorder from healthy controls, by use of multi-scale structural magnetic resonance imaging analyses. On account of the limited knowledge of the neural basis of PMDD and our aim to provide a comprehensive analysis of grey matter structure in this disorder, voxel-wise whole-brain analyses were conducted as exploratory investigations, in combination with complementary region-of-interest (ROI)-based analyses targeting the regions reported to be altered in PMDD (mostly task-related functional findings [6]). In line with the hypothesis of an impaired top-down inhibitory process involving limbic brain structures in premenstrual dysphoric disorder, differences in grey matter morphology were expected within regions of corticolimbic networks.

## Participants and methods

### Participants

This study was based on two neuroimaging datasets collected at Uppsala University Hospital (site 1) and Umeå University Centre for Functional Brain Imaging (site 2), Sweden. In total, 94 women with PMDD (62 from site 1 and 32 from site 2), and 43 healthy controls (11 from site 1 and 32 from site 2) with regular menstrual cycles, and Swedish-speaking, were recruited by advertisement in local newspapers, boards, social media, and students’ websites. Exclusion criteria were: steroid hormone (including oral contraceptives) and psychotropic treatments in the previous three months, breast-feeding, pregnancy, presence of other ongoing psychiatric disorders, severe medical conditions, and contraindications for MRI. Monitoring of the menstrual cycle phase was confirmed by serum progesterone and oestradiol concentrations. The authors assert that all procedures contributing to this work comply with the ethical standards of the relevant national and institutional committees on human experimentation and with the Helsinki Declaration of 1975, as revised in 2008. All procedures were approved by the Ethical Review Board in Uppsala (Dnr. 2016/184 and 2016/312) and Umeå (2016-111-31M, 2017-266-32M), and written informed consent was obtained from all participants.

PMDD diagnosis, according to DSM-5 criteria, was confirmed by use of daily prospective symptom ratings during two consecutive menstrual cycles. Women reported on the Daily Record of Severity of Problems (DRSP) scale using a smartphone application or a web platform. PMDD was defined as >50% increase in at least five of eleven symptoms (at least one core PMDD symptom) between the follicular (day 6 to 12) and luteal phase (day −7 to −1) [18]. Percent increase was calculated as [(mean luteal phase scores – mean follicular phase scores)/mean follicular phase scores) × 100]. Psychiatric comorbidity was ruled out by the MINI-International Neuropsychiatric Interview [19].

### Hormone analyses

Venous blood samples were collected from each participant at the beginning of every MR session. Oestradiol and progesterone serum concentrations from site 1 were quantified by liquid chromatography–tandem mass spectrometry at the Core Facility of Metabolomics, University of Bergen. At site 2, corresponding analyses were performed using standard methods at the central laboratory at Umeå University Hospital.

### MR acquisition

Most of the participants (97%) were scanned during the late luteal phase (day −5 to 0) and the mid-luteal phase (day −10 to −6). Due to technical issues, two women were scanned in the early luteal phase (day −11 and day −13), and one was scanned in the early follicular phase (day 2) but was kept in the analyses as she was still highly symptomatic. Acquisition of high-resolution T1-weighted images at site 1 was conducted with a 3.0 Tesla whole-body scanner (Achieva dStream, Philips Medical Systems, Best, The Netherlands) equipped with a 32-channel head coil, using an MPRAGE sequence (TR = 8.3 ms, TE = 3.8 ms, 256 × 256 matrix size, flip angle = 8°, 220 transversal slices, acquisition time: 3:50 min). Resulting images have a 0.94 × 0.94 × 1 mm^3^ voxel size. Acquisition of high-resolution T1-weighted images at site 2 was performed with a 3.0 Tesla Discovery MR750 scanner (General Electric, Madison, WI, USA) equipped with a 32-channel head coil, using a 3D fast spoiled gradient echo sequence (TR = 8.2 ms, TE = 3.2 ms, 512 × 512 matrix size, flip angle = 12°, 176 transversal slices, acquisition time: 8:11 min). Resulting images have a 0.48 × 0.48 × 1 mm^3^ voxel size. In order to account for the difference in scanning protocols, the site variable was included in the statistical analyses as a confounding factor.

### Voxel-based morphometry

The MR pre-processing and analyses were run using the Statistical Parametric Mapping software (SPM12, Welcome Trust Centre for Neuroimaging, University College London, UK) implemented in MATLAB R2019b (MathWorks, Natick, MA, USA). First, all images were manually reoriented using the coordinate of the anterior commissure as origin (0, 0, 0). Using the segment routine of SPM12, the reoriented images were spatially normalized into the Montreal Neurological Institute (MNI) space, corrected for intensity variations, and segmented into tissue compartments [20]. A modulation process was applied to the grey matter probability maps to compensate for the effects of spatial normalization on volumetric data. Finally, modulated grey matter probability maps were smoothed using an 8-mm full-width half-maximum (FWHM) Gaussian kernel. The voxel size was 1.5 × 1.5 × 1.5 mm^3^.

A quality assessment procedure including a visual inspection and an automated quality control using the CAT12 toolbox (http://dbm.neuro.uni-jena.de/cat) was employed to detect outliers. The average total GMV was 0.74 ± 0.05 L and the mean total intracranial volume (TIV) was 1.51 ± 0.11 L.

### Surface-based morphometry

For SBM pre-processing and analyses, we used the automated CAT12 pre-processing pipeline including a projection-based thickness estimation [21], along with partial volume correction and correction for sulcal blurring and sulcal asymmetries. A gyrification index was extracted based on absolute mean curvature [22]. In addition, cortical complexity and sulcal depth measures were extracted. For inter-subject comparisons, the surface meshes were re-parameterized into a common coordinate system using spherical maps [23]. Finally, all surface measures were resampled and smoothed with a Gaussian kernel, of 15 mm (FWHM) for cortical thickness and 20 mm (FWHM) for the other parameters, using the recommended settings. The automated CAT12 quality control module for surface data was used to detect outliers.

### Statistical analyses

Due to the unequal group sizes, all statistical analyses were run according to non-parametric models. In order to account for the nuisance variance of regressors of non-interest, TIV, age and site were included as confounding covariates. VBM and SBM analyses were conducted using complementary exploratory (hypothesis-free) whole-brain and hypothesis-driven ROI approaches.

VBM exploratory analyses of GMV consisted of whole-brain voxel-wise group comparisons conducted within a mask of grey matter defined by an absolute threshold set at 0.2. In addition, we defined 12 ROIs based on the results of the previous literature on PMDD [6], including the bilateral anterior cingulate cortex (ACC), amygdala, hippocampus, parahippocampal gyrus (PHG), fusiform gyrus (FuG), orbitofrontal cortex (OFC), the inferior, middle and superior frontal gyri (IFG, MFG and SFG), insula, putamen and cerebellum. The ROIs were defined according to the Automatic Anatomical Labelling (AAL) atlas, using the PickAtlas toolbox in SPM12. Similar to the exploratory analyses, we performed voxel-wise group comparisons analyses, within the bilateral anatomical masks for both the cortical and subcortical ROIs (amygdala, putamen and hippocampus). Sub-regional post hoc analyses of the amygdala (i.e., medial and lateral amygdala) and putamen (i.e., ventral-posterior, ventral-anterior, dorsal-posterior and dorsal-anterior) were carried out, based on the Melbourne subcortex atlas [24].

We assessed group differences in brain surface parameters using exploratory whole-brain vertex-wise group comparisons. In addition, regional mean surface data were extracted using CAT12, based on the FreeSurfer Desikan/Killiany atlas [25]. Among the available surface parcellated regions (excluding subcortical areas and cerebellum), 26 unilateral ROIs from the left and right hemispheres corresponded to the volumetric ROIs defined above and were analysed, including the caudal ACC, rostral ACC, insula, PHG, FuG and eight prefrontal regions (superior frontal, pars opercularis, pars orbitalis, pars triangularis, rostral middle frontal, caudal middle frontal, lateral orbitofrontal and medial orbitofrontal).

Voxel- and vertex-wise VBM and SBM analyses were conducted using the non-parametric permutation-based threshold-free cluster enhancement (TFCE) method [26]. TFCE was applied using 10,000 permutations and a significance threshold of *p* < 0.05 Family Wise Error (FWE)-corrected. Cohen’s *d* effect size maps were generated using the CAT12 toolbox.

Based on previous literature showing asymmetry in grey matter structure [27] and brain function related to emotion regulation [28], the analyses based on surface ROI-extracted data were carried out for each hemisphere separately, using the Statistical Package for the Social Sciences (SPSS) version 26. Non-parametric Quade’s ANCOVAs were conducted to assess group differences in ROI-extracted data while considering TIV, age, and site as confounding covariates. The significance threshold was set at *p* < 0.05. Bonferroni correction was applied for the volumetric analyses (12 bilateral ROI masks, *p*_Bonferroni_ < 0.0042), and the surface analyses (26 unilateral ROIs, *p*_Bonferroni_ < 0.0019).

In order to investigate whether grey matter metrics in regions that displayed significant differences between women with PMDD and controls relate to PMDD symptom severity, we carried out post hoc partial correlation analyses in a sub-sample of women with PMDD for which the DRSP scores of the scanning month were available (*n* = 55). Using TFCE in SPM with 10,000 permutations and a significance threshold of *p* < 0.05 FWE-corrected, partial correlations between grey matter metrics and the DRSP scores were tested within masks combining the significant clusters highlighting group differences. Specifically, the total DRSP score and core PMDD symptoms (depression, irritability, affective lability and anxiety) DRSP scores were investigated. To account for the nuisance variance of regressors of non-interest, TIV, age, and BMI were included as confounding covariates due to their potential influence on volumetric and surface measures [29–31].

Additional whole-brain exploratory analyses further assessing the influence of variables of non-interest (age, prior psychiatric history and menstrual cycle phase) on the findings were conducted. The main analyses exploring the group differences in GMV and cortical thickness was repeated (i) in a model adjusting for prior psychiatric diagnoses, (ii) in a sub-sample where groups did not differ in age, and (iii) in a sub-sample excluding the three participants that were scanned either in the early follicular or early luteal phase.

### Multivariate pattern classification analysis (MVPA)

In addition, we performed pattern classification to distinguish women with PMDD from controls based on each structural measure. To that end, we used the MVPANI toolbox (http://funi.tmu.edu.cn) [32] implemented in Matlab. All participants were divided into 10 folds, resulting in 13 participants in the first 9 folds and 14 participants in the last 4 folds. The proportion of patients and controls was balanced to remain stable across folds. A leave-one-fold-out cross-validation procedure was used to compute an average classification accuracy across all folds. The significance of this average classification was calculated using 5000 permutations due to the high computational power required, based on the null distribution obtained by randomly shuffling the labels of the participants in the training dataset. The resulting *p*-values reflect the proportion of permutations yielding greater or equivalent accuracy compared to the actual classification accuracy, from the total number of permutations. We further explored the specific contribution of our 12 predefined VBM ROIs in the classification accuracy based on GMV. For this ROI-based approach, the same model was used, and the left and right masks of our ROIs were tested separately (except for the cerebellum, due to its medial location). A Bonferroni correction was applied to the ROI-based statistics, bringing the significance threshold at *p* < 0.0022 (22 unilateral ROIs + 1 bilateral cerebellum ROI = 23 tests). For all classification models, classification accuracy, specificity and sensitivity, receiver operating characteristic (ROC) curves and areas under the curve (AUCs) were computed.

We also performed a searchlight classification analysis based on GMV, to obtain a finer localization of the regions contributing the most to the classification accuracy. To do so, the same model was run throughout the GMV maps using spheres of 4 mm diameter. For this analysis, the p-values were computed using 1000 permutations, due to the high computational power required.

## Results

### Participant characteristics

Demographic and psychometric characteristics of the participants are presented in Table 1. Due to technical issues (*n* = 4), brain tumour (*n* = 1), and claustrophobia (*n* = 1), a total of 6 participants (5 PMDD, 1 control) were excluded from the analyses, which thus included 89 women with PMDD and 42 controls. This sample size provided statistical power (1−*β* = 0.8) to detect medium to large effects (Cohen’s *d* > 0.5). The two groups did not differ in terms of BMI, TIV, serum oestradiol and progesterone concentration, assessment timing, or menstrual cycle length. However, women with PMDD were on average slightly older than controls and more often reported prior mood disorders (Table 1). The following VBM and SBM findings remained virtually the same when adjusting for prior psychiatric diagnoses (Table S1), age (Table S2) and menstrual cycle phase at scanning (Table S3).

**Table 1:** Participant characteristics.

	HC (*n* = 42) Mean ± SD or *n* (%)	PMDD (*n* = 89) Mean ± SD or *n* (%)
Age (years)	28 ± 6	33 ± 7*
BMI	23.8 ± 3.8	24.1 ± 4.0
TIV (L)	1.52 ± 0.11	1.50 ± 0.10
Menstrual cycle length (days)	28 ± 2	28 ± 2
PMDD duration (years)^a^	–	11 ± 7
Psychiatric history	7 (16.6%)	34 (38.2%)*
Depressive disorder	6 (85.7%)	27 (79.4%)
Anxiety disorder	1 (14.3%)	6 (17.6%)
Eating disorder	0	4 (11.8%)
Oestradiol (pmol/L)	434.5 ± 47.4	411.1 ± 239.8
Progesterone (nmol/L)	18.7 ± 15.0	21.9 ± 15.3
Luteal phase scanning day	−4 ± 2	−4 ± 3
Total DRSP score	38.6 ± 13.9	61.8 ± 18.2*

Differences between the groups were assessed using Mann–Whitney test for continuous variables, and Fischer Exact test for categorical variables.

*BMI* body mass index, *DRSP* daily record of severity of problems, *SD* standard deviation, *TIV* total intracranial volume, *PMDD* premenstrual dysphoric disorder, *HC* healthy controls.

*Significant group difference at *p* < 0.05.

^a^Data only available for a subset of 58 women with PMDD.

### Smaller grey matter volume in PMDD: voxel-based morphometry

**Fig. 1:**
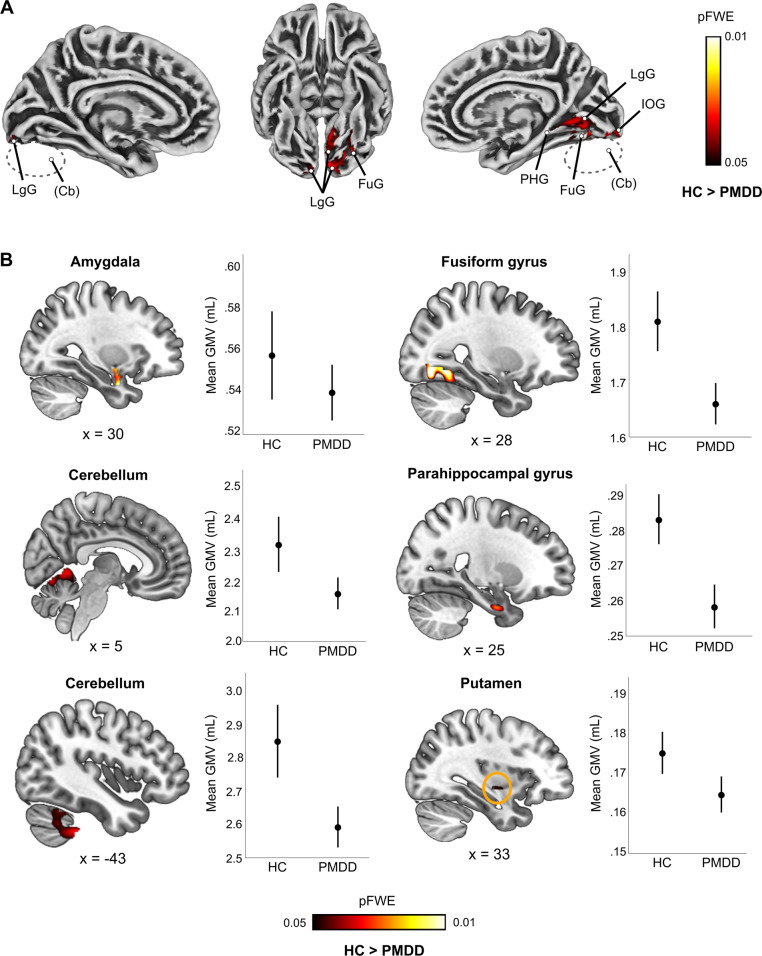
Group difference in grey matter volume between women with premenstrual dysphoric disorder and healthy controls (*p*_FWE_ < 0.05, TFCE). **A** Surface representation of the significant clusters showing lower grey matter volume in women with PMDD compared to healthy controls across the whole brain. **B** Sagittal slice overlays and plots illustrating lower grey matter volume in women with PMDD compared to healthy controls within ROIs (TFCE, *p* < 0.05, FWE-corrected). Localization of the brain slices along the x axis is given by the Montreal Neurological Institute coordinates below each slice. The plots illustrate the average raw grey matter volume within the significant clusters resulting from the voxel-wise ROI analyses, in the PMDD and the control groups. The error bars represent 95% confidence interval. No significant group difference in the opposite direction was found. Cb cerebellum, FuG fusiform gyrus, FWE Family Wise Error correction, GMV grey matter volume, IOG inferior occipital gyrus, LgG lingua gyrus, PHG parahippocampal gyrus, TFCE Threshold Free Cluster Enhancement, PMDD premenstrual dysphoric disorder, HC healthy controls.

Women with PMDD displayed smaller GMV compared to healthy controls at the whole-brain level. The difference was driven by seven clusters covering parts of the cerebellum, lingual, fusiform, inferior occipital and parahippocampal gyri (*p*_FWE_ < 0.05; 0.45 < *d* < 0.76) (Fig. 1A, Table S4).

In addition, as illustrated in Fig. 1B, the ROI analyses suggested that women with PMDD had smaller right amygdala (0.34 < *d* < 0.55) and putamen volumes (0.47 < *d* < 0.53) (*p*_FWE_ < 0.05), although these results did not survive Bonferroni correction (Table S5). Sub-regional analyses suggest that these effects primarily involved the right lateral amygdala (*p*_FWE_ < 0.05; 0.33 < *d* < 0.55), and the ventral putamen (*p*_FWE_ < 0.05; 0.33 < *d* < 0.54) (Table S5).

### Thinner cortices in PMDD: surface-based morphometry

**Fig. 2:**
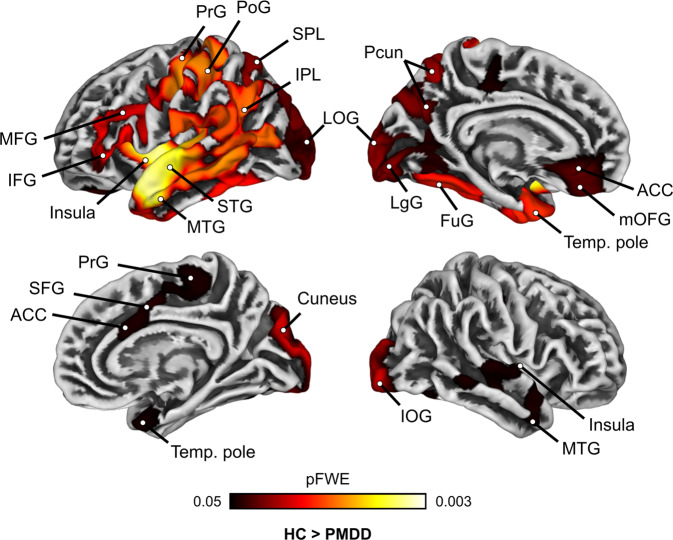
Whole-brain group comparison of cortical thickness between women with premenstrual dysphoric disorder and healthy controls (*p*_FWE_ < 0.05, TFCE). Surface representation of the significant clusters showing thinner cortex in women with PMDD compared to healthy controls across the whole brain. No significant group difference in the opposite direction was found. ACC anterior cingulate cortex, FuG fusiform gyrus, FWE Family Wise Error correction, IFG inferior frontal gyrus, IOG inferior occipital gyrus, IPL inferior parietal lobule, LgG lingua gyrus, LOG lateral occipital gyrus, MFG middle frontal gyrus, mOFG medial orbitofrontal gyrus, MTG middle temporal gyrus, PHG parahippocampal gyrus, PoG postcentral gyrus, PrG precentral gyrus, SFG superior frontal gyrus, SPL superior parietal lobule, STG superior temporal gyrus, Temp temporal, TFCE Threshold Free Cluster Enhancement, PMDD premenstrual dysphoric disorder, HC healthy controls.

Across the whole brain, we identified six large clusters where women with PMDD had thinner cortices than controls (*p*_FWE_ < 0.05; 0.20 < *d* < 0.74) (Table S6, Fig. 2). The largest difference was found in the left hemisphere, in a cluster covering the temporal pole, middle- and superior temporal gyri, insula, inferior- and middle frontal gyri, rostral ACC, medial OFC, paracentral lobule, inferior- and superior parietal lobules, precuneus, FuG, lingual and lateral occipital gyri. A similar group difference was found within the right hemisphere, in five smaller clusters covering the cuneus and inferior occipital gyrus, temporal pole, insula, caudal ACC, SFG and paracentral lobule (Fig. 2). No differences in gyrification, sulcal depth and cortical complexity were found between women with PMDD and controls at the *p*_FWE_ < 0.05 threshold.

Subsequent ROI-based analyses conducted on mean extracted surface measures yielded similar patterns of group differences in cortical thickness. However, they did not survive correction for multiple testing across all ROIs (Fig. S1, Tables S7–S10).

### Grey matter structure distinguishes the PMDD brain: pattern classification analyses

**Fig. 3:**
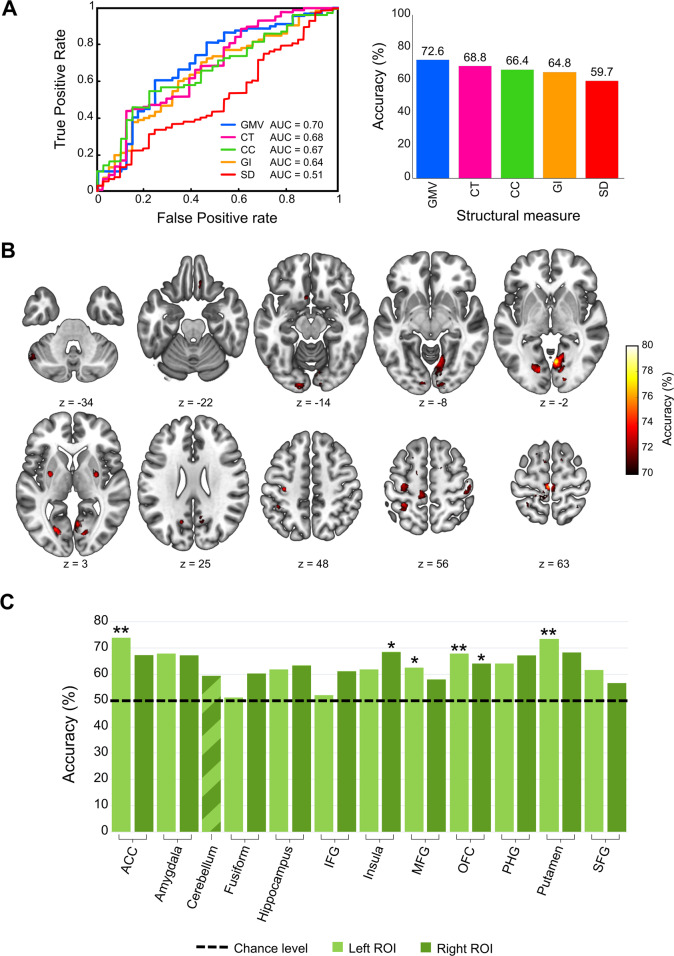
Classification results. **A** Pattern classification of women with premenstrual dysphoric disorder and healthy controls based on the five structural measures over the whole-brain. Receiver operating characteristic (ROC) curves and corresponding area under curve (AUC) are displayed for each structural measure in the left graph. Mean accuracies of classification are presented in the right graph. **B** Classification accuracy map of the searchlight MVPA (voxel-wise) based on the GMV images. The centres of searchlight spheres where higher-than-chance level accuracies were found under the threshold *p* < 0.001 (uncorrected) are depicted in red-yellow on axial brain slices. The classification accuracy under this threshold ranged from 58.0 to 78.6% across the brain. For visualization purposes, only the accuracies >70% are displayed. **C** ROI-based classification based on GMV images results. The chance-level threshold (50% accuracy, 0.5 AUC) is indicated by the horizontal black dotted line. *p < 0.05, uncorrected for multiple testing **p < 0.05 Bonferroni-corrected (*p*_Bonferroni_ < 0.0022). GMV grey matter volume, CT cortical thickness, CC cortical complexity, GI gyrification index, SD sulcal depth, ACC anterior cingulate cortex, Fusiform fusiform gyrus, IFG inferior frontal gyrus, MFG middle frontal gyrus, SFG superior frontal gyrus, OFC orbitofrontal cortex, PHG parahippocampal gyrus.

Classification analyses based on each of the structural grey matter measures indicated that GMV was the best measure for distinguishing women with PMDD from healthy controls, with an accuracy of 72.6% (Fig. 3A). In comparison, the accuracies of cortical thickness (68.6%), cortical complexity (66.4%), gyrification index (64.8%) and sulcal depth (59.8%), as measures distinguishing women with PMDD, were lower (Fig. 3A, Table S11).

Furthermore, we investigated the specific brain areas contributing to the GMV classification accuracy. The searchlight MVPA highlighted a number of regions providing a classification accuracy higher than 70% (Fig. 3B). After correcting for multiple testing across the spheres, only the right lingual gyrus and the left paracentral lobule remained significant (Fig. S2). The ROI-based classification of women with PMDD and controls further points to a significant influence of GMV within the left ACC and putamen, with an accuracy of about 73–74%, *p* < 0.05, Bonferroni-corrected (Fig. 3C, Table S12).

### Association between differential grey matter measures and symptom severity

Among the women with PMDD that reported on symptoms severity during the scanning month (*n* = 55), we found no significant correlation between the thickness of regions showing significant group differences and any of the tested DRSP scores assessing premenstrual symptoms. Likewise, no significant association was found between the GMV of regions that differed between the groups and the DRSP scores. At a trend level, however, we observed negative correlations between the GMV of cerebellum areas and the severity of depression and affective lability scores (*p*_FWE_ < 0.1, Fig. S3, Table S13).

## Discussion

**Fig. 4:**
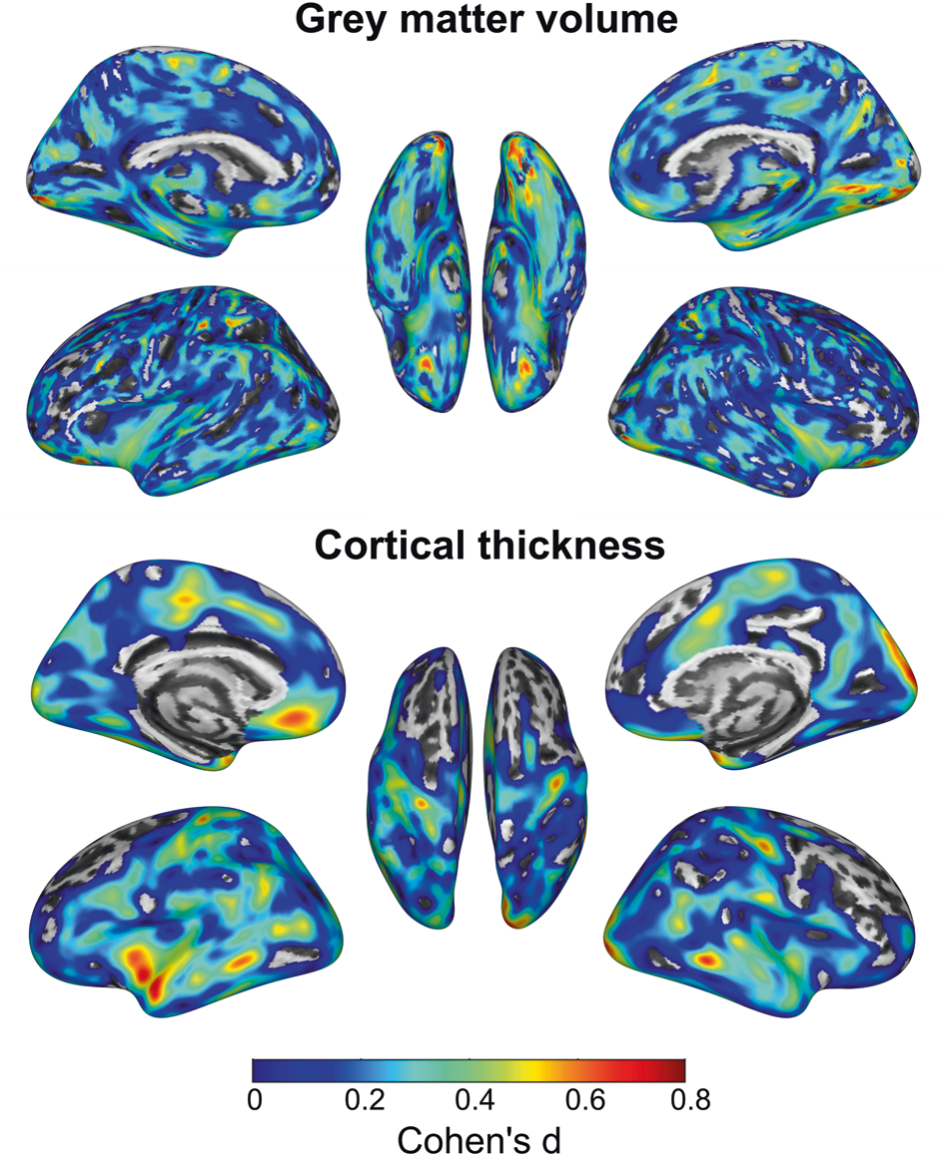
Effect sizes. Illustration of the effect sizes (Cohen’s *d*) for the difference between women with premenstrual dysphoric disorder and healthy controls in grey matter volume cortical thickness. Views of the left and right hemispheres are shown on the left and right sides, respectively. The middle brain representation shows the bottom view in the upper panel and the top view in the lower panel. Medium to large effect sizes are depicted in orange-red. Effect size maps were generated using the CAT12 toolbox (http://dbm.neuro.uni-jena.de/cat).

These results indicate that women with PMDD display noticeable differences in grey matter structure compared with controls. More specifically, women with PMDD present smaller GMV, especially in the lingual, fusiform, inferior occipital and cerebellar areas, and subcortical regions such as the amygdala and putamen. The distinction of women with PMDD was confirmed by multivariate pattern classification analyses, where lower GMV in the right lingual gyrus, and left paracentral lobule, ACC and putamen was the best predictor for the classification of women with PMDD, with an accuracy of 73–78%. Among the surface metrics, global cortical thickness was the best classifier, with an accuracy of 68%. In line with this, women with PMDD displayed thinner cortices in widespread brain regions compared with controls. Remarkably, the effect sizes for these structural differences were moderate to large (Fig. 4). Our findings suggest a neurobiological susceptibility in women with PMDD, which we hypothesize may predispose or participate in the development of their condition.

Evidence for structural differences characterizing emotional disorders comes from meta-analyses conducted on major depressive and anxiety disorders. Importantly, the present findings are consistent with these studies regarding the direction of the effect, that is pointing to smaller GMV [13, 14, 33], and thinner cortices in patients compared to controls [11, 12]. Moreover, regarding the localization of the effects, the regions we report on partially overlap with the regions showing thinner cortex in individuals with depression or anxiety (i.e., temporal regions, the insula, ACC, OFC, MFG and superior parietal lobule), and those showing smaller GMV in individuals with comorbid depression and anxiety (i.e. the amygdala and PHG) [13], two core symptoms experienced by women with PMDD. Thus, according to our findings, in the luteal phase, the brain structure of women with PMDD may differ from controls beyond these previously reported areas, further involving the GMV of cerebellar regions, putamen and inferior occipital cortex, and the cortical thickness of frontal, parietal, and occipital regions.

Notably, these regions overlap with the brain areas highlighted by task-based functional neuroimaging studies of PMDD (primarily targeting emotional processing), namely the amygdala, ventral striatum, insula, ACC, PFC, pre and post central gyri, parietal lobules and cerebellum [6]. Among these, the amygdala, ventral striatum, insula, medial PFC and OFC constitute key hubs in emotional networks [34]. Thus, differential grey matter structure in these regions could influence the affective symptoms characterizing PMDD. Furthermore, we found smaller cerebellar volumes in women with PMDD, in both anterior and posterior regions covering functional areas that have been related to motor, cognitive and affective processing [35]. Our findings are also in line with the localization of regions previously shown to functionally and/or structurally vary across the menstrual cycle, such as the amygdala, insula, ACC, PFC, pre and post central gyri, parietal lobules, temporal and occipital areas and cerebellum [7], and thus probably influenced by fluctuations of ovarian hormones.

Interestingly, the lingual gyrus stood out from the present analyses, as the one region highlighted by the VBM, SBM and the multivariate pattern analyses. Smaller activation in the lingual gyrus has been found in women with PMDD compared to controls, during a facial emotion recognition task [36], while the lone use of a priori ROIs and small sample sizes in other fMRI studies [37–39] may explain the lack of previous findings implicating this region in visual emotional processing in PMDD. Together with the FuG, the lingual gyrus is involved in face processing [40]. Early research suggests that it shares direct connections with the limbic system [41], possibly through the inferior longitudinal fasciculus [42]. In fact, the interaction between mood regulation and visual information processing have been demonstrated by a study on depression suggesting that abnormal visual processing interferes with the recruitment of attention neural networks in MDD patients, potentially impacting cognitive functions [43]. On the structural level, variations in cortical thickness [44], GMV [45] and surface area [46] of the lingual gyrus have been associated with depression and anxiety. Thus, we posit that PMDD may be characterized by similar alterations in top-down control regions influencing the limbic system and visual processing areas, together leading to severe premenstrual affective and cognitive symptoms in the luteal phase. In line with this, Dan and colleagues [47] recently showed that women with PMDD exhibit poorer temporal-occipital functional connections compared to controls across the menstrual cycle, connections which mediated the relationship between difficulties in emotion regulation and PMDD diagnosis. The structural differences here depicted between women with PMDD and controls, including the volume and thickness of occipital regions and the thickness of temporal regions, might relate to these functional findings.

The data-driven multivariate analyses further suggest differences in GMV in the putamen, as well as the ACC and paracentral regions where thinner cortices were detected. While the ACC and ventral striatum are hypothesized to play a role in the pathophysiology of PMDD as key regions of emotional networks [6], the involvement of the paracentral area is less clear. Petersen et al. [48] reported reduced reactivity of this region during an emotion regulation task in women with PMDD, whereas a recent resting-state MRI study found a hypo-connectivity of this brain area with temporal cortices in women with PMDD, which did not relate to difficulties in emotion regulation [47]. Of note, the paracentral lobule has been previously linked to mood disorders, as highlighted by a meta-analysis on GMV in major depression [49]. Besides, a reduced GMV of the paracentral region was found to be involved in the subjective negative perception (consequences, emotional representations and catastrophizing) of pain, specifically in women in chronic pain compared to men [50]. Although this region appears to participate in both mood regulation and pain perception, which are of relevance to PMDD symptomatology, further investigations are warranted to define the role of the paracentral area in the pathophysiology of PMDD. Remarkably, our findings of differential grey matter structure of the lingual gyrus and paracentral area in PMDD are in line with the previously suggested role of the primary sensorimotor cortex in emotional regulation [51].

Together, our findings overlap with the functional hubs or several well-defined brain networks including the salience network (dorsal ACC, anterior insula, putamen and extended amygdala), the executive control network (dorsal and ventrolateral PFC, parietal lobules) [52], the default mode network (medial and dorsal PFC, ACC, middle temporal gyrus, PHG, parietal lobules) [53, 54] and visual pathways (occipital, lingual and fusiform gyri) [55]. This constitutes further indication for the involvement of distinct and multiple networks in the PMDD pathophysiology, possibly relating to the variety of affective, cognitive and physical symptoms experienced by women with PMDD on a monthly basis [1]. Although we did not find any significant correlation between the differential grey matter measures and symptom severity in the sub-sample of women with PMDD for which we had the data, we found negative correlations at a trend level between GMV in the cerebellum and the premenstrual scores of depression and affective lability. These trends are in line with the recently reported negative association between depression-related PMDD symptoms and the GMV of the amygdala [56]. Nevertheless, these findings call for replication studies including large samples of participants and prospective symptom ratings to establish whether regions that show differential grey matter structure in PMDD are associated with symptom severity, as qualitative and quantitative relationships may be proxies of trait vs. state features of PMDD, respectively.

Notwithstanding the extensive use of VBM in brain research, the biological underpinnings of GMV changes remain largely unknown. MRI grey matter measures are widely assumed to depend on neuronal cell bodies, dendrites, non-myelinated axons, and glial cells. Studies on rodents points to a positive association of VBM-based measures with dendritic spine density [57] and volume [58], with most of the GMV measures being accounted for by neuronal axons collaterals and dendrites, extracellular space, neuron soma and astrocytes [57, 58]. Moreover, ovarian hormones have been shown to be involved in rapid fluctuations of dendritic density in the amygdala across the oestrous cycle [59, 60], as well as in depression- and anxiety-like behaviour [60]. Thus, the smaller GMV found here in women with PMDD could be explained by tissue shrinkage, change in nucleus volume, cellular density, and dendritic volume and density, while the thinner cortices could involve differences in neuronal and glial cells [61].

The current study displays a number of strengths compared to the neuroimaging literature on PMDD, such as the use of multi-scale structural brain analyses, a rather large sample of women with PMDD, the confirmation of menstrual cycle phase through both cycle mapping and hormonal assessment, and the control for confounding factors (age and TIV). All factors that likely explain the discrepancy between the present and previous findings [8–10]. In addition, whole-brain analyses were conducted as exploratory investigations, in combination with complementary ROI-based analyses, thus offering the possibility to provide results that can be used as the basis for future research and detect both rather large and extended effects, and smaller effects in regions that are more likely to be affected in PMDD. As a technical limitation, the currently available tools allowing multivariate pattern analyses for classification based on neuroimaging data do not enable us to investigate the regional contribution of surface measures in a vertex-wise manner. Furthermore, with a sample size of 131 women included in the analyses, our volumetric and surface findings reached a statistical power up to 0.99 (Cohen’s *d* = 0.75). However, yet significant at the *p*_FWE_ < 0.05 threshold, our ROI voxel-based volumetric findings in the right amygdala and sub-regions of the amygdala and putamen (0.32 < *d* < 0.54) should be considered with caution, as such smaller effects would require a larger sample [62]. In addition, considering the low contrast between tissues within subcortical structures in MR images and its potential impact on automated segmentation pipelines [63], these subcortical results further call for replication. Finally, it is important to note the difference in psychiatric history between the groups, though it is expected, as the differentially structured regions partly overlap with those found in studies on related disorders. After repeating the analysis while controlling for previous psychiatric diagnoses, age difference between the groups, and menstrual cycle phase, the results remained unchanged (Tables S1–S3). Further research investigating grey matter structure throughout the menstrual cycle in women with PMDD and controls should clarify whether these structural differences are luteal phase-specific or represent long-lasting changes.

In conclusion, the present findings point to PMDD-specific grey matter structure in regions of corticolimbic networks, in line with the hypothesis of an impaired top-down inhibitory circuit involving limbic structures in PMDD. Furthermore, the results include widespread cortical regions and cerebellar areas, suggesting the involvement of distinct networks in PMDD pathophysiology. These effects prominently involved GMV and cortical thickness, as further highlighted by multivariate pattern classification analyses, while the global architecture of the cortex did not differ between women with PMDD and controls. Such differences in brain structure may help explaining the variations in brain function previously reported in women with this condition.

## Supplementary information

supplement
