# Supplementary material for: Differential grey matter structure in women with premenstrual dysphoric disorder: evidence from brain morphometry and data-driven classification
Source: Transl Psychiatry. 2022 Jun 15;12:250. doi: 10.1038/s41398-022-02017-6 (PMC9200862; doi:10.1038/s41398-022-02017-6)

**Supplementary material**

**Table S1:** **Whole-brain group comparison of grey matter structure between women with premenstrual dysphoric disorder and healthy controls, controlling for previous psychiatric diagnoses.**

|  | **Cluster size** | | **p_FWE_** | | **MNI coordinates** | | | **AAL** |
| --- | --- | --- | --- | --- | --- | --- | --- | --- |
|  |  |  |  |  | **x** | **y** | **z** |  |
| ***HC > PMDD*** | | | | | | | | |
| *Grey matter volume* | | | | | | | | |
|  | 532 | | 0.033 | | 18 | -80 | -12 | Lingual_R |
|  |  | | 0.033 | | 24 | -74 | -11 | Fusiform_R |
|  | 309 | | 0.037 | | 12 | -62 | -5 | Lingual_R |
|  |  | | 0.044 | | 9 | -51 | -5 | Cerebelum_4_5_R |
|  | 37 | | 0.047 | | -14 | -87 | -15 | Lingual_L |
| *Cortical thickness* | | | |  | | | | |
|  | 16126 | | 0.003 | | -51 | 7 | -24 | Temporal_Mid_L |
|  |  | | 0.004 | | -58 | -9 | -3 | Temporal_Sup_L |
|  |  | | 0.008 | | -46 | -29 | 61 | Postcentral_L |
|  |  | | 0.011 | | -47 | -54 | 29 | Angular_L |
|  |  | | 0.011 | | -31 | -8 | -36 | Fusiform_L |
|  | 1470 | | 0.012 | | -55 | -33 | 50 | Parietal_Inf_L |
|  |  | | 0.016 | | 9 | -97 | 15 | Cuneus_R |
|  |  | | 0.016 | | 29 | -97 | -5 | Occipital_Inf_R |
|  |  | | 0.049 | | 39 | -82 | 23 | Occipital_Mid_R |
|  | 1051 | | 0.037 | | 5 | -6 | 49 | Supp_Motor_Area_R |
|  | 1381 | | 0.039 | | 58 | -22 | -2 | Temporal_Sup_R |
|  |  | | 0.049 | | 53 | 11 | -29 | Temporal_Pole_Mid_R |
|  | 973 | | 0.042 | | 55 | -18 | 19 | Rolandic_Oper_R |
|  | 574 | | 0.045 | | 10 | -56 | 18 | Precuneus_R |
|  | 524 | | 0.048 | | 24 | -35 | 59 | Postcentral_R |
|  | 92 | | 0.048 | | 39 | -36 | -21 | Fusiform_R |
| ***HC < PMDD*** | |  | | | | | | |
| *Grey matter volume* | | N.S. | | | | | | |
| *Cortical thickness* | | N.S. | | | | | | |

The main whole-brain analysis was repeated to investigate the impact of the “previous psychiatric diagnoses” covariate on the findings. We replicated our initial findings of greater GMV and cortical thickness in controls compared to women with PMDD (p_FWE_<0.05). The opposite group comparison did not yield any significant result, in line with our original findings. Redundant peaks within clusters are not presented. Abbreviations: AAL, Automatic Anatomical Labelling atlas; FWE, Family Wise Error correction; PMDD, premenstrual dysphoric disorder; HC, healthy controls; L, left; R, right; N.S., non-significant.

**Table S2: Whole-brain group comparison of grey matter structure between women with premenstrual dysphoric disorder and healthy controls matched for age.**

|  | **Cluster size** | | **p_FWE_** | | **MNI coordinates** | | | **AAL** |
| --- | --- | --- | --- | --- | --- | --- | --- | --- |
|  |  |  |  |  | **x** | **y** | **z** |  |
| ***HC > PMDD*** | | | | | | | | |
| *Grey matter volume* | | | | | | | | |
|  | 904 | | 0.052 | | 12 | -87 | -12 | Lingual_R |
|  |  | | 0.059 | | 24 | -75 | -11 | Fusiform_R |
|  |  | | 0.094 | | 26 | -92 | -12 | Occipital_Inf_R |
|  | 475 | | 0.063 | | 14 | -60 | -5 | Lingual_R |
|  |  | | 0.087 | | 11 | -51 | -5 | Cerebelum_4_5_R |
|  | 153 | | 0.081 | | -12 | -87 | -15 | Lingual_L |
|  | 26 | | 0.095 | | -20 | -74 | -5 | Lingual_L |
| *Cortical thickness* | | | |  | | | | |
|  | 2865 | | 0.040 | | -42 | -0 | -19 | Temporal Pole Sup L |
|  |  | | 0.044 | | -48 | 5 | -27 | Temporal_Mid_L |
|  |  | | 0.050 | | -59 | -7 | -3 | Temporal_Sup_L |
|  |  | | 0.085 | | -33 | -1 | 15 | Insula_L |
|  |  | | 0.086 | | -32 | -12 | -33 | Fusiform_L |
|  | 960 | | 0.085 | | -19 | -93 | 25 | Occipital_Sup_L |
|  |  | | 0.089 | | -5 | -81 | 39 | Cuneus_L |
|  |  | | 0.093 | | -23 | -100 | -8 | Occipital_Inf_L |
|  | 228 | | 0.092 | | -53 | -61 | 10 | Temporal_Mid_L |
|  | 29 | | 0.100 | | -9 | -65 | 23 | Calcarine_L |
| ***HC < PMDD*** | |  | | | | | | |
| *Grey matter volume* | | N.S. | | | | | | |
| *Cortical thickness* | | N.S. | | | | | | |

The main analysis was repeated in a sub-sample for which there was no significant age difference (N=109, 32 controls and 77 women with PMDD), in order to account for the 5-year age difference between the initial groups, and to explore how it impacted the findings. We replicated our initial findings of greater GMV and cortical thickness in controls compared to women with PMDD, however at a more lenient statistical threshold (p_FWE_<0.1). The opposite group comparison did not yield any significant result, in line with our original findings. Redundant peaks within clusters are not presented. Cluster size is given in voxels for the VBM results, and vertices for the SBM results. Abbreviations: AAL, Automatic Anatomical Labelling atlas; FWE, Family Wise Error correction; PMDD, premenstrual dysphoric disorder; HC, healthy controls; L, left; R, right; N.S., non-significant.

**Table S3: Whole-brain group comparison of grey matter structure between women with premenstrual dysphoric disorder and healthy controls, excluding the three participants scanned in the early follicular and early luteal phase.**

|  | **Cluster size** | | **p_FWE_** | | **MNI coordinates** | | | **AAL** |
| --- | --- | --- | --- | --- | --- | --- | --- | --- |
|  |  |  |  |  | **x** | **y** | **z** |  |
| ***HC > PMDD*** | | | | | | | | |
| *Grey matter volume* | | | | | | | | |
|  |  | | 2294 | | 11 | -83 | -12 | Lingual_R |
|  |  |  |  |  | 26 | -74 | -11 | Fusiform_R |
|  |  | |  |  | 9 | -51 | -5 | Cerebelum_4_5_R |
|  |  | |  |  | 5 | -69 | -12 | Vermis_6 |
|  |  |  |  |  | -2 | -59 | -6 | Vermis_4_5 |
|  |  | |  |  | 26 | -92 | -12 | Occipital_Inf_R |
|  |  | | 224 | | -14 | -87 | -15 | Lingual_L |
|  |  | | 174 | | 36 | -87 | -11 | Occipital_Inf_R |
|  |  | | 20 | | 41 | -68 | -20 | Fusiform_R |
|  |  | | 2 | | -18 | -77 | -6 | Lingual_L |
|  |  | | 1 | | -20 | -74 | -5 | Lingual_L |
| *Cortical thickness* | | | |  | | | | |
|  | 10457 | | 0.007 | | -43 | 2 | -19 | Temporal_Pole_Sup_L |
|  |  | | 0.008 | | -51 | 7 | -24 | Temporal_Mid_L |
|  |  | | 0.011 | | -48 | -5 | -5 | Temporal_Sup_L |
|  |  | | 0.014 | | -35 | -15 | 46 | Precentral_L |
|  |  | | 0.015 | | -46 | -29 | 61 | Postcentral_L |
|  |  | | 0.016 | | -50 | -57 | 35 | Parietal_Inf_L |
|  |  | | 0.022 | | -35 | -37 | -22 | Fusiform_L |
|  |  | | 0.025 | | -41 | 41 | 1 | Frontal_Inf_Tri_L |
|  |  | | 0.033 | | -8 | -61 | 50 | Precuneus_L |
|  | 708 | | 0.027 | | 9 | -97 | 15 | Cuneus_R |
|  |  | | 0.028 | | 4 | -88 | 15 | Cuneus_L |
|  |  | | 0.030 | | 24 | -94 | 17 | Occipital_Sup_R |
|  |  | | 0.039 | | 34 | -80 | -16 | Occipital_Inf_R |
|  | 1379 | | 0.038 | | -12 | -97 | -3 | Calcarine_L |
|  |  | | 0.039 | | -6 | -80 | -13 | Lingual_L |
|  |  | | 0.042 | | -11 | -78 | 36 | Cuneus_L |
|  | 212 | | 0.042 | | -7 | 27 | -12 | Frontal_Med_Orb_L |
|  | 157 | | 0.048 | | 6 | 8 | 41 | Cingulum_Mid_R |
|  | 89 | | 0.048 | | -14 | -24 | 40 | Cingulum_Mid_L |
|  | 37 | | 0.049 | | 35 | -18 | 44 | Precentral_R |
| ***HC < PMDD*** | |  | | | | | | |
| *Grey matter volume* | | N.S. | | | | | | |
| *Cortical thickness* | | N.S. | | | | | | |

The main analysis was repeated while excluding the one participant scanned in the early follicular phase (day 2) and two participants scanned in the early luteal phase (day -13 and day -11), resulting in 86 women with PMDD and 42 controls. We replicated our initial findings of greater GMV and cortical thickness in controls compared to women with PMDD (p_FWE_<0.05). The opposite group comparison did not yield any significant result, in line with our original findings. Redundant peaks within clusters are not presented. Cluster size is given in voxels for the VBM results, and vertices for the SBM results. Abbreviations: AAL, Automatic Anatomical Labelling atlas; FWE, Family Wise Error correction; PMDD, premenstrual dysphoric disorder; HC, healthy controls; L, left; R, right; N.S., non-significant.

**Table S4: Whole-brain group comparison of grey matter volume between women with premenstrual dysphoric disorder and healthy controls.**

|  | **Cluster size (voxels)** | | **p_FWE_** | **MNI coordinates** | | | **Cohen’s d range** | **AAL** |
| --- | --- | --- | --- | --- | --- | --- | --- | --- |
|  |  |  |  | **x** | **y** | **z** |  |  |
| ***HC > PMDD*** | |  | | | | | | |
|  | 2095 | | 0.024 | 12 | -87 | -9 | 0.45 – 0.75 | Lingual R |
|  |  |  | 0.026 | 26 | -74 | -11 |  | Fusiform R |
|  |  |  | 0.031 | 9 | -51 | -5 |  | Cerebellum 4 5 R |
|  |  |  | 0.038 | 5 | -69 | -11 |  | Vermis 6 |
|  |  |  | 0.041 | -2 | -59 | -6 |  | Vermis 4 5 |
|  |  |  | 0.041 | -5 | -57 | -3 |  | Cerebellum 4 5 L |
|  |  |  | 0.045 | 26 | -92 | -12 |  | Occipital Inf R |
|  | 228 | | 0.036 | -14 | -87 | -15 | 0.54 – 0.76 | Lingual L |
|  | 81 | | 0.041 | 36 | -87 | -11 | 0.51 – 0.74 | Occipital Inf R |
|  | 9 | | 0.050 | 24 | -6 | -32 | 0.60 – 0.63 | Parahippocampus R |
| ***HC < PMDD*** | | |  |  |  | N.S. |  |  |

Redundant peaks within clusters are not presented. Abbreviations: AAL, Automatic Anatomical Labelling atlas; FWE, Family Wise Error correction; MNI, Montreal Neurological Institute; TFCE, Threshold Free Cluster Enhancement; PMDD, premenstrual dysphoric disorder; HC, healthy controls; L, left; R, right.

**Table S5: Region-of-Interest group comparison of grey matter volume between women with premenstrual dysphoric disorder and healthy controls (TFCE).**

| **Region of Interest** | **Cluster size (voxels)** | **p_FWE_ corrected (TFCE)** | **MNI coordinates** | | | | | **Cohen’s d range** | **AAL** |
| --- | --- | --- | --- | --- | --- | --- | --- | --- | --- |
|  |  |  | **x** | **y** | | | **z** |  |  |
| ***HC > PMDD*** | | | | | | | | | |
| ACC | N.S. | | | | | | | | |
| Amygdala | 297 | 0.008 | 30 | | 3 | -29 | | 0.34 - 0.55 | Amygdala R |
| Lateral | 215 | 0.012 | 26 | | -3 | -29 | | 0.33 - 0.55 | Amygdala R |
|  | *22* | *0.085* | *-30* | | *2* | *-18* | | *0.32 - 0.38* | *Amygdala L* |
| *Medial* | *18* | *0.084* | *29* | | *-9* | *-12* | | *0.40 - 0.47* | *Amygdala R* |
| Cerebellum | 1282 | 0.026 | 9 | | -51 | -5 | | 0.39 - 0.63 | Cerebellum 4 5 R |
|  |  | 0.030 | 14 | | -83 | -17 | |  | Cerebellum 6 R |
|  |  | 0.031 | -2 | | 59 | -3 | |  | Vermis 4 5 |
|  |  | 0.033 | 5 | | -69 | -11 | |  | Vermis 6 |
|  | 1508 | 0.028 | -42 | | -41 | -47 | | 0.39 - 0.62 | Cerebellum 8 L |
|  |  | 0.039 | -47 | | -56 | -30 | |  | Cerebellum Crus1 L |
|  |  | 0.045 | -39 | | -54 | -24 | |  | Cerebellum 6 L |
|  | 93 | 0.041 | -15 | | -83 | -17 | | 0.47 - 0.65 | Cerebellum 6 L |
| FuG | 913 | 0.005 | 26 | | -74 | -11 | | 0.37 – 0.68 | Fusiform R |
|  | 11 | 0.049 | 38 | | -14 | -30 | | 0.44 – 0.48 | Fusiform R |
| Hippocampus | N.S. | | | | | | | | |
| IFG | N.S. | | | | | | | | |
| Insula | N.S. | | | | | | | | |
| MFG | N.S. | | | | | | | | |
| *OFC* | *147* | *0.058* | *-21* | | *23* | *-17* | | *0.51 – 0.66* | *Frontal Inf Orb L* |
|  | *95* | *0.063* | *23* | | *26* | *-14* | | *0.50 – 0.67* | *Frontal Inf Orb R* |
| PHG | 134 | 0.020 | 24 | | -6 | -32 | | 0.46 - 0.64 | Parahippocampus R |
| Putamen | 38 | 0.043 | 30 | | 8 | -8 | | 0.48 – 0.53 | Putamen R |
|  | 51 | 0.044 | 32 | | -11 | -9 | | 0.47 - 0.52 | Putamen R |
|  | *74* | *0.089* | *-30* | | *-15* | *6* | | *0.40 - 0.46* | *Putamen L* |
| Ant.Ventral | 38 | 0.028 | 29 | | 9 | -8 | | 0.41 - 0.54 | Putamen R |
|  | *16* | *0.087* | *-26* | | *11* | *-8* | | *0.34 - 0.40* | *Putamen L* |
| Post.Ventral | 278 | 0.009 | 32 | | -11 | -11 | | 0.34 - 0.52 | Putamen R |
|  | 338 | 0.019 | -30 | | -15 | 8 | | 0.33 - 0.47 | Putamen L |
| *Ant.Dorsal* | *23* | *0.085* | *30* | | *8* | *-6* | | *0.42 - 0.50* | *Putamen R* |
| Post. Dorsal | N.S. | | | | | | | | |
| SFG | N.S. | | | | | | | | |
| ***HC < PMDD*** | N.S. | | | | | | | | |

Redundant peaks within clusters are not presented. Trend-level results were visualized at a threshold of p_FWE_ < 0.10 and are shown in italic. The results did not survive Bonferroni correction for multiple testing across the 12 bilateral ROIs (p_Bonferroni_ = 0.0042). Abbreviations: AAL, Automatic Anatomical Labelling atlas; Ant. Dorsal, anterior part of the dorsal putamen; Ant. Ventral, anterior part of the ventral putamen; FWE, Family Wise Error correction; Lateral, lateral part of the amygdala; Medial, medial part of the amygdala; MNI, Montreal Neurological Institute; N.S., Non-significant; Post. Dorsal, posterior part of the dorsal putamen; Post. Ventral, posterior part of the ventral putamen; TFCE, Threshold Free Cluster Enhancement; PMDD, premenstrual dysphoric disorder; HC, healthy controls; L, left; R, right.

**Table S6: Whole-brain group comparison of surface parameters between women with premenstrual dysphoric disorder and healthy controls.**

|  | **Cluster size (vertices)** | | | **p_FWE_** | **MNI coordinates** | | | **Cohen’s d range** | **AAL** |
| --- | --- | --- | --- | --- | --- | --- | --- | --- | --- |
|  |  |  |  |  | **x** | **y** | **z** |  |  |
| ***HC > PMDD*** | | |  | | | | | | |
| *Cortical thickness* | | |  | | | | | | |
|  | 13384 | | | 0.004 | -51 | 7 | -24 | 0.20 – 0.74 | Temporal Mid L |
|  |  |  |  | 0.004 | -38 | 3 | -7 |  | Insula L |
|  |  |  |  | 0.010 | -46 | -29 | 61 |  | Postcentral L |
|  |  |  |  | 0.010 | -45 | -17 | 59 |  | Precentral L |
|  |  |  |  | 0.011 | -47 | -54 | 29 |  | Angular L |
|  |  |  |  | 0.014 | -55 | -42 | 45 |  | Parietal Inf L |
|  | 882 | | | 0.021 | 9 | -97 | 15 | 0.33 – 0.72 | Cuneus R |
|  |  |  |  | 0.022 | 29 | -97 | -5 |  | Occipital Inf R |
|  | 120 | | | 0.044 | 67 | -38 | -6 | 0.41 – 0.68 | Temporal Mid R |
|  | 143 | | | 0.045 | 23 | 11 | -35 | 0.45 – 0.63 | Temporal Pole Sup R |
|  | 916 | | | 0.045 | 53 | 11 | -29 | 0.32 – 0.50 | Temporal Pole Mid R |
|  |  |  |  | 0.045 | 46 | -13 | 4 |  | Insula R |
|  | 200 | | | 0.045 | 35 | -17 | 42 | 0.42 – 0.61 | Precentral R |
| *Gyrification index* | | |  | | | | | | |
| N.S. | | | | | | | | | |
| *Sulcal depth* | | |  | | | | | | |
| N.S. | | | | | | | | | |
| *Cortical complexity* | | |  | | | | | | |
| N.S. | | | | | | | | | |
| ***HC < PMDD*** | | N.S. | | | | | | | |

Redundant peaks within clusters are not presented. Abbreviations: AAL, Automatic Anatomical Labelling atlas; FWE, Family Wise Error correction; MNI, Montreal Neurological Institute; N.S., Non-significant; TFCE, Threshold Free Cluster Enhancement; PMDD, premenstrual dysphoric disorder; HC, healthy controls; L, left; R, right.


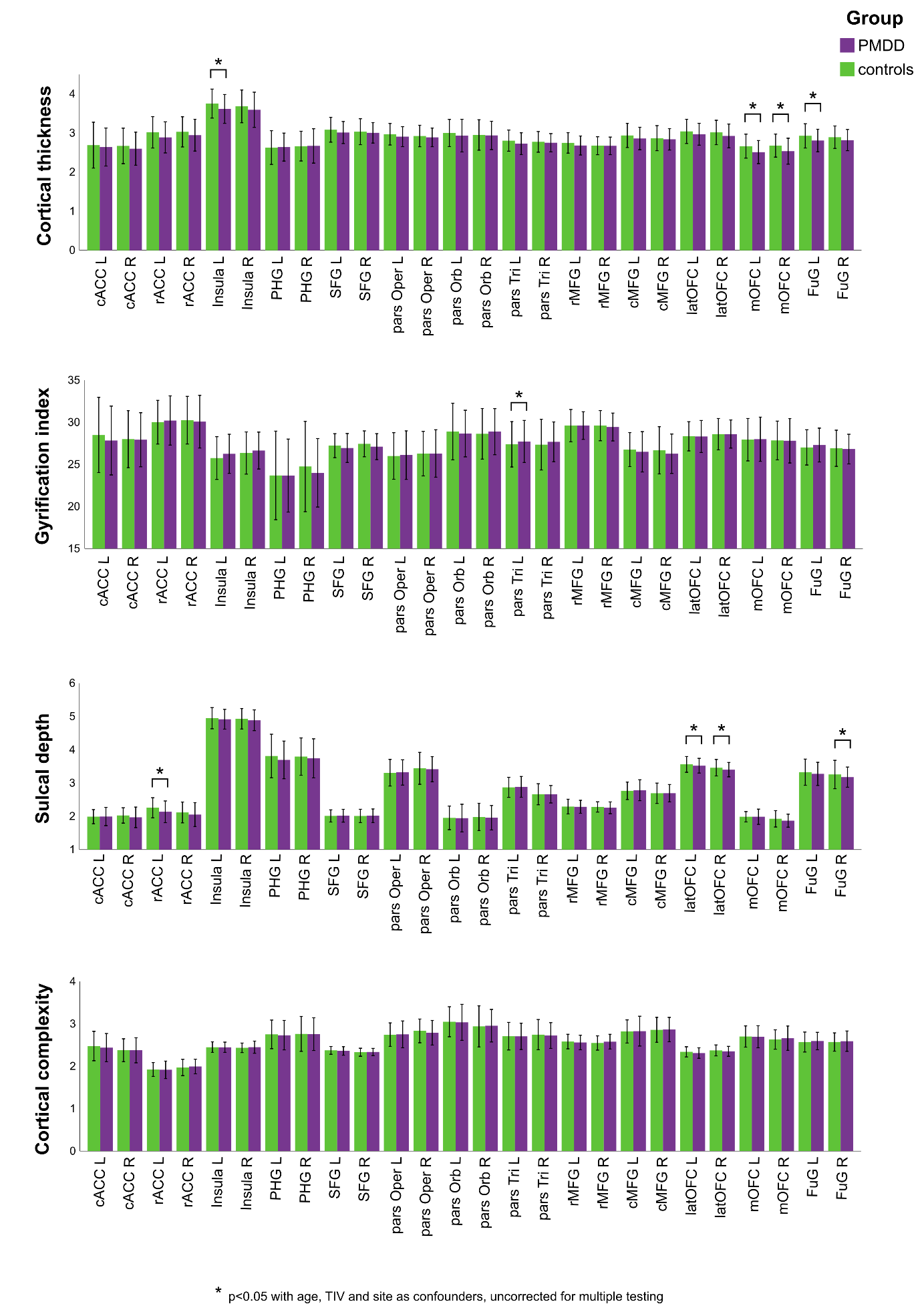
**Figure S1.** **Group differences in surface metrics between women with premenstrual dysphoric disorder and healthy controls within regions-of-interest.** Mean surface metrics within the cortical ROIs in the two groups. Error bars indicate 2 standard deviations. * p < 0.05 after correcting for TIV, age and site, uncorrected for multiple testing. Abbreviations: cACC, caudal anterior cingulate cortex; rACC, rostral anterior cingulate cortex; FuG, fusiform gyrus; cMFG, caudal middle frontal gyrus; rMFGrostral middle frontal gyrus; latOFC, lateral orbitofrontal cortex; mOFC, medial orbitofrontal cortex; parsOrb, pars orbitalis; parsOper, pars opercularis; parsTri, pars triangularis; PHG, parahippocampal gyrus; SFG, superior frontal gyrus; TIV, total intracranial volume; PMDD, premenstrual dysphoric disorder; L, left; R, right.

| **Region-of-interest** | **Mean (SD) HC** | **Mean (SD) PMDD** | **F ratio** | **p-value** | **Cohen’s d** |
| --- | --- | --- | --- | --- | --- |
| Caudal ACC L | 2.69 (0.29) | 2.64 (0.24) | 0.35 | 0.852 | 0.19 |
| Caudal ACC R | 2.67 (0.23) | 2.60 (0.21) | 2.40 | 0.123 | 0.32 |
| Rostral ACC L | 3.02 (0.20) | 2.89 (0.20) | 3.76 | 0.055 | 0.65 |
| Rostral ACC R | 3.03 (0.19) | 2.95 (0.20) | 0.22 | 0.641 | 0.41 |
| **Insula L*** | **3.75 (0.18)** | **3.62 (0.18)** | **5.62** | **0.019** | **0.72** |
| Insula R | 3.68 (0.21) | 3.59 (0.22) | 1.23 | 0.270 | 0.42 |
| Parahippocampus L | 2.63 (0.22) | 2.64 (0.18) | 1.08 | 0.302 | 0.05 |
| Parahippocampus R | 2.66 (0.19) | 2.67 (0.22) | 0.43 | 0.515 | 0.05 |
| SFG L | 3.08 (0.16) | 3.01 (0.14) | 0.10 | 0.750 | 0.47 |
| SFG R | 3.03 (0.17) | 3.00 (0.13) | 0.79 | 0.375 | 0.20 |
| Pars Opercularis L | 2.97 (0.14) | 2.91 (0.13) | 1.71 | 0.193 | 0.44 |
| Pars Opercularis R | 2.92 (0.14) | 2.89 (0.12) | 0.96 | 0.330 | 0.23 |
| Pars Orbitalis L | 3.00 (0.17) | 2.93 (0.21) | 0.18 | 0.668 | 0.37 |
| Pars Orbitalis R | 2.95 (0.19) | 2.94 (0.18) | 0.16 | 0.898 | 0.05 |
| Pars Triangularis L | 2.80 (0.13) | 2.73 (0.14) | 2.35 | 0.128 | 0.52 |
| Pars Triangularis R | 2.77 (0.13) | 2.75 (0.12) | 0.27 | 0.604 | 0.16 |
| Rostral MFG L | 2.75 (0.14) | 2.68 (0.12) | 0.28 | 0.597 | 0.54 |
| Rostral MFG R | 2.67 (0.11) | 2.67 (0.11) | 1.34 | 0.249 | 0.00 |
| Caudal MFG L | 2.94 (0.15) | 2.86 (0.14) | 0.30 | 0.585 | 0.55 |
| Caudal MFG R | 2.87 (0.16) | 2.84 (0.14) | 0.50 | 0.480 | 0.20 |
| Lateral OFC L | 3.04 (0.15) | 2.97(0.14) | 0.25 | 0.619 | 0.48 |
| Lateral OFC R | 3.01 (0.16) | 2.92 (0.15) | 1.72 | 0.192 | 0.58 |
| **Medial OFC L*** | **2.66 (0.15)** | **2.51 (0.15)** | **7.23** | **0.008** | **1.00** |
| **Medial OFC R*** | **2.68 (0.15)** | **2.53 (0.17)** | **4.06** | **0.046** | **0.94** |
| **Fusiform L*** | **2.93 (0.15)** | **2.81 (0.14)** | **4.84** | **0.030** | **0.83** |
| Fusiform R | 2.89 (0.14) | 2.82 (0.14) | 0.82 | 0.367 | 0.50 |

**Table S7.** **Group difference in cortical thickness between women with premenstrual dysphoric disorder and healthy controls within regions-of-interest.**

The mean cortical thickness and standard deviation (SD) within the regions-of-interest (ROIs) in the two groups, and the statistics from non-parametric Quade’s ANCOVAs are presented. * p < 0.05 after correcting for total intracranial volume, age and site, uncorrected for multiple testing. Effect sizes, as illustrated by the Cohen’s d values, where calculated according to the formula Cohen's d = (Mean_Group2_ - Mean_Group1_) ⁄ SD_pooled_, where SD_pooled_ = √((SD_1_^2^ + SD_2_^2^) ⁄ 2). Abbreviations: ACC, anterior cingulate cortex; Fusiform, fusiform gyrus; MFG, middle frontal gyrus; OFC, orbitofrontal cortex; PHG, parahippocampal gyrus; SFG, superior frontal gyrus; PMDD, premenstrual dysphoric disorder; HC, healthy controls; L, left; R, right.

**Table S8.** **Group difference in gyrification index between women with premenstrual dysphoric disorder and healthy controls within regions-of-interest.**

| **Region-of-interest** | **Mean (SD) HC** | **Mean (SD) PMDD** | **F ratio** | **p-value** | **Cohen’s d** |
| --- | --- | --- | --- | --- | --- |
| Caudal ACC L | 28.49 (2.23) | 27.84 (2.04) | 2.33 | 0.129 | 0.30 |
| Caudal ACC R | 28.00 (1.69) | 27.92 (1.6) | 0.32 | 0.572 | 0.05 |
| Rostral ACC L | 30.01 (1.30) | 30.21 (1.46) | 1.44 | 0.232 | 0.14 |
| Rostral ACC R | 30.25 (1.41) | 30.07 (1.56) | 0.39 | 0.535 | 0.12 |
| Insula L | 25.75 (1.27) | 26.26 (1.16) | 0.40 | 0.530 | 0.42 |
| Insula R | 26.35 (1.25) | 26.64 (1.10) | 1.32 | 0.253 | 0.25 |
| Parahippocampus L | 23.69 (2.63) | 23.67 (2.17) | 0.25 | 0.616 | 0.01 |
| Parahippocampus R | 24.75 (2.68) | 24.00 (2.03) | 0.51 | 0.477 | 0.31 |
| SFG L | 27.22 (0.71) | 26.95 (0.86) | 0.36 | 0.550 | 0.34 |
| SFG R | 27.44 (0.77) | 27.10 (0.77) | 1.16 | 0.283 | 0.01 |
| Pars Opercularis L | 26.00 (1.38) | 26.10 (1.44) | 1.04 | 0.310 | 0.07 |
| Pars Opercularis R | 26.28 (1.32) | 26.30 (1.41) | 0.53 | 0.469 | 0.01 |
| Pars Orbitalis L | 28.90 (1.68) | 28.66 (1.38) | 0.27 | 0.604 | 0.16 |
| Pars Orbitalis R | 28.63 (1.50) | 28.88 (1.37) | 0.91 | 0.343 | 0.17 |
| **Pars Triangularis L*** | **27.39 (1.35)** | **27.72 (1.25)** | **4.48** | **0.036** | **0.25** |
| Pars Triangularis R | 27.34 (1.50) | 27.68 (1.18) | 2.05 | 0.154 | 0.25 |
| Rostral MFG L | 29.60 (0.96) | 29.61 (0.82) | 0.31 | 0.580 | 0.01 |
| Rostral MFG R | 29.60 (0.90) | 29.44 (0.83) | 0.54 | 0.465 | 0.18 |
| Caudal MFG L | 26.75 (1.01) | 26.50 (1.20) | 0.74 | 0.391 | 0.22 |
| Caudal MFG R | 26.67 (1.40) | 26.27 (1.17) | 1.39 | 0.241 | 0.31 |
| Lateral OFC L | 28.34 (0.86) | 28.32 (0.95) | 0.09 | 0.770 | 0.02 |
| Lateral OFC R | 28.59 (0.93) | 28.60 (0.83) | 0.00 | 0.972 | 0.01 |
| Medial OFC L | 27.94 (1.26) | 27.99 (1.31) | 0.00 | 0.948 | 0.04 |
| Medial OFC R | 27.85 (1.15) | 27.81 (1.32) | 0.01 | 0.938 | 0.03 |
| Fusiform L | 27.02 (1.04) | 27.30 (1.00) | 0.57 | 0.452 | 0.27 |
| Fusiform R | 26.91 (1.07) | 26.82 (0.88) | 0.17 | 0.682 | 0.09 |

The mean gyrification index and standard deviation (SD) within the regions-of-interest (ROIs) in the two groups, and the statistics from non-parametric Quade’s ANCOVAs are presented. * p < 0.05 after correcting for total intracranial volume, age and site, uncorrected for multiple testing. Effect sizes, as illustrated by the Cohen’s d values, where calculated according to the formula Cohen's d = (Mean_Group2_ - Mean_Group1_) ⁄ SD_pooled_, where SD_pooled_ = √((SD_1_^2^ + SD_2_^2^) ⁄ 2). Abbreviations: ACC, anterior cingulate cortex; Fusiform, fusiform gyrus; MFG, middle frontal gyrus; OFC, orbitofrontal cortex; PHG, parahippocampal gyrus; SFG, superior frontal gyrus; PMDD, premenstrual dysphoric disorder; HC, healthy controls; L, left; R, right.

**Table S9.** **Group difference in sulcal depth between women with premenstrual dysphoric disorder and healthy controls within regions-of-interest.**

| **Region-of-interest** | **Mean (SD) HC** | **Mean (SD) PMDD** | **F ratio** | **p-value** | **Cohen’s d** |
| --- | --- | --- | --- | --- | --- |
| Caudal ACC L | 1.98 (0.11) | 1.99 (0.14) | 0.13 | 0.720 | 0.08 |
| Caudal ACC R | 2.03 (0.11) | 1.97 (0.15) | 3.14 | 0.079 | 0.46 |
| **Rostral ACC L*** | **2.26 (0.15)** | **2.13 (0.16)** | **8.20** | **0.005** | **0.84** |
| Rostral ACC R | 2.11 (0.16) | 2.05 (0.18) | 1.83 | 0.178 | 0.35 |
| Insula L | 4.95 (0.16) | 4.92 (0.15) | 1.34 | 0.250 | 0.19 |
| Insula R | 4.93 (0.15) | 4.88 (0.16) | 1.62 | 0.206 | 0.32 |
| Parahippocampus L | 3.81 (0.33) | 3.69 (0.28) | 2.84 | 0.094 | 0.39 |
| Parahippocampus R | 3.79 (0.28) | 3.74 (0.29) | 0.69 | 0.408 | 0.17 |
| SFG L | 2.01 (0.09) | 2.02 (0.10) | 0.01 | 0.910 | 0.10 |
| SFG R | 2.01 (0.10) | 2.02 (0.10) | 0.10 | 0.748 | 0.10 |
| Pars Opercularis L | 3.31 (0.20) | 3.32 (0.19) | 0.01 | 0.930 | 0.05 |
| Pars Opercularis R | 3.44 (0.24) | 3.41 (0.19) | 0.18 | 0.670 | 0.14 |
| Pars Orbitalis L | 1.95 (0.18) | 1.95 (0.21) | 0.04 | 0.848 | 0.00 |
| Pars Orbitalis R | 1.98 (0.20) | 1.96 (0.18) | 1.17 | 0.281 | 0.10 |
| Pars Triangularis L | 2.87 (0.15) | 2.88 (0.16) | 0.01 | 0.902 | 0.06 |
| Pars Triangularis R | 2.66 (0.15) | 2.66 (0.13) | 0.40 | 0.529 | 0.00 |
| Rostral MFG L | 2.29 (0.11) | 2.28 (0.10) | 0.01 | 0.914 | 0.09 |
| Rostral MFG R | 2.28 (0.08) | 2.25 (0.09) | 1.41 | 0.238 | 0.35 |
| Caudal MFG L | 2.76 (0.13) | 2.78 (0.16) | 0.23 | 0.635 | 0.14 |
| Caudal MFG R | 2.69 (0.15) | 2.69 (0.13) | 0.07 | 0.789 | 0.00 |
| **Lateral OFC L*** | **3.56 (0.12)** | **3.52 (0.11)** | **5.45** | **0.021** | **0.35** |
| **Lateral OFC R*** | **3.46 (0.12)** | **3.40 (0.11)** | **6.00** | **0.016** | **0.52** |
| Medial OFC L | 1.99 (0.08) | 1.99 (0.11) | 0.14 | 0.709 | 0.00 |
| Medial OFC R | 1.93 (0.12) | 1.87 (0.10) | 2.54 | 0.113 | 0.54 |
| Fusiform L | 3.33 (0.20) | 3.27 (0.17) | 0.85 | 0.357 | 0.32 |
| **Fusiform R*** | **3.26 (0.21)** | **3.18 (0.15)** | **6.09** | **0.015** | **0.44** |

The mean sulcal depth and standard deviation (SD) within the regions-of-interest (ROIs) in the two groups, and the statistics from non-parametric Quade’s ANCOVAs are presented. * p < 0.05 after correcting for total intracranial volume, age and site, uncorrected for multiple testing. Effect sizes, as illustrated by the Cohen’s d values, where calculated according to the formula Cohen's d = (Mean_Group2_ - Mean_Group1_) ⁄ SD_pooled_, where SD_pooled =_ √((SD_1_^2^ + SD_2_^2^) ⁄ 2). Abbreviations: ACC, anterior cingulate cortex; Fusiform, fusiform gyrus; MFG, middle frontal gyrus; OFC, orbitofrontal cortex; PHG, parahippocampal gyrus; SFG, superior frontal gyrus; PMDD, premenstrual dysphoric disorder; HC, healthy controls; L, left; R, right.

| **Region-of-interest** | **Mean (SD) HC** | **Mean (SD) PMDD** | **F ratio** | **p-value** | **Cohen’s d** |
| --- | --- | --- | --- | --- | --- |
| Caudal ACC L | 2.48 (0.17) | 2.44 (0.17) | 0.26 | 0.611 | 0.23 |
| Caudal ACC R | 2.38 (0.13) | 2.38 (0.15) | 0.32 | 0.571 | 0.00 |
| Rostral ACC L | 1.92 (0.08) | 1.92 (0.10) | 0.01 | 0.942 | 0.00 |
| Rostral ACC R | 1.97 (0.10) | 1.99 (0.09) | 0.35 | 0.556 | 0.21 |
| Insula L | 2.45 (0.06) | 2.45 (0.06) | 0.00 | 0.952 | 0.00 |
| Insula R | 2.44 (0.06) | 2.45 (0.07) | 0.00 | 0.960 | 0.15 |
| Parahippocampus L | 2.75 (0.17) | 2.73 (0.17) | 0.06 | 0.804 | 0.12 |
| Parahippocampus R | 2.76 (0.20) | 2.76 (0.19) | 0.27 | 0.607 | 0.00 |
| SFG L | 2.37 (0.05) | 2.36 (0.05) | 2.90 | 0.091 | 0.20 |
| SFG R | 2.33 (0.05) | 2.34 (0.04) | 0.02 | 0.898 | 0.22 |
| Pars Opercularis L | 2.74 (0.14) | 2.75 (0.16) | 0.04 | 0.839 | 0.07 |
| Pars Opercularis R | 2.83 (0.14) | 2.79 (0.14) | 2.31 | 0.131 | 0.29 |
| Pars Orbitalis L | 3.05 (0.18) | 3.04 (0.21) | 0.01 | 0.927 | 0.05 |
| Pars Orbitalis R | 2.94 (0.24) | 2.96 (0.19) | 0.66 | 0.417 | 0.09 |
| Pars Triangularis L | 2.71 (0.16) | 2.71 (0.15) | 0.01 | 0.937 | 0.00 |
| Pars Triangularis R | 2.75 (0.18) | 2.72 (0.15) | 0.14 | 0.709 | 0.18 |
| Rostral MFG L | 2.58 (0.09) | 2.56 (0.09) | 0.04 | 0.846 | 0.22 |
| Rostral MFG R | 2.55 (0.08) | 2.58 (0.09) | 2.82 | 0.095 | 0.35 |
| Caudal MFG L | 2.82 (0.14) | 2.83 (0.18) | 0.01 | 0.937 | 0.06 |
| Caudal MFG R | 2.86 (0.15) | 2.87 (0.14) | 0.05 | 0.816 | 0.07 |
| Lateral OFC L | 2.34 (0.06) | 2.31 (0.06) | 1.90 | 0.171 | 0.50 |
| Lateral OFC R | 2.37 (0.06) | 2.35 (0.06) | 1.93 | 0.168 | 0.33 |
| Medial OFC L | 2.70 (0.12) | 2.70 (0.13) | 0.01 | 0.927 | 0.00 |
| Medial OFC R | 2.63 (0.11) | 2.66 (0.14) | 3.71 | 0.056 | 0.24 |
| Fusiform L | 2.57 (0.12) | 2.59 (0.10) | 1.32 | 0.252 | 0.18 |
| Fusiform R | 2.57 (0.11) | 2.59 (0.12) | 0.06 | 0.807 | 0.17 |

**Table S10.** **Group difference in cortical complexity between women with premenstrual dysphoric disorder and healthy controls within regions-of-interest.**

The mean cortical complexity and standard deviation (SD) within the regions-of-interest (ROIs) in the two groups, and the statistics from non-parametric Quade’s ANCOVAs are presented. No significant difference between the groups was found, even at the uncorrected level. Effect sizes, as illustrated by the Cohen’s d values, where calculated according to the formula Cohen's d = (Mean_Group2_ - Mean_Group1_) ⁄ SD_pooled_, where SD_pooled =_ √((SD_1_^2^ + SD_2_^2^) ⁄ 2). Abbreviations: ACC, anterior cingulate cortex; Fusiform, fusiform gyrus; MFG, middle frontal gyrus; OFC, orbitofrontal cortex; PHG, parahippocampal gyrus; SFG, superior frontal gyrus; PMDD, premenstrual dysphoric disorder; HC, healthy controls; L, left; R, right.

**Table S11: Whole-brain classification results across surface measures.**

| **Modality** | **MVPA Accuracy (%)** | **Specificity** | **Sensitivity** | **AUC** | **p-value** |
| --- | --- | --- | --- | --- | --- |
| Cortical thickness | 68.79 | 47.79 | 78.65 | 0.68 | 0.003 |
| Gyrification index | 64.83 | 38.09 | 77.53 | 0.64 | 0.018 |
| Sulcal depth | 59.67 | 30.95 | 73.03 | 0.51 | 0.177 |
| Cortical complexity | 66.43 | 33.33 | 82.02 | 0.67 | 0.006 |

Multivariate Pattern Classification Analysis (MVPA) results for the global grey matter volume, cortical thickness, gyrification index, sulcal depth and cortical complexity. The p-value indicates the significance of the original MVPA, and was obtained from non-parametric permutation testing using 5000 permutations. AUC, area under curve.

**Figure S2: Searchlight MVPA results after Family-Wise Error correction.** Classification accuracy map of the searchlight Multivariate Pattern Classification Analysis, MVPA (voxel-wise) based on the grey matter volume images (p < 0.05; Family-Wise Error (FWE)-corrected). The centers of searchlight spheres where higher-than-chance level accuracies were found are depicted in red-yellow on axial brain slices.
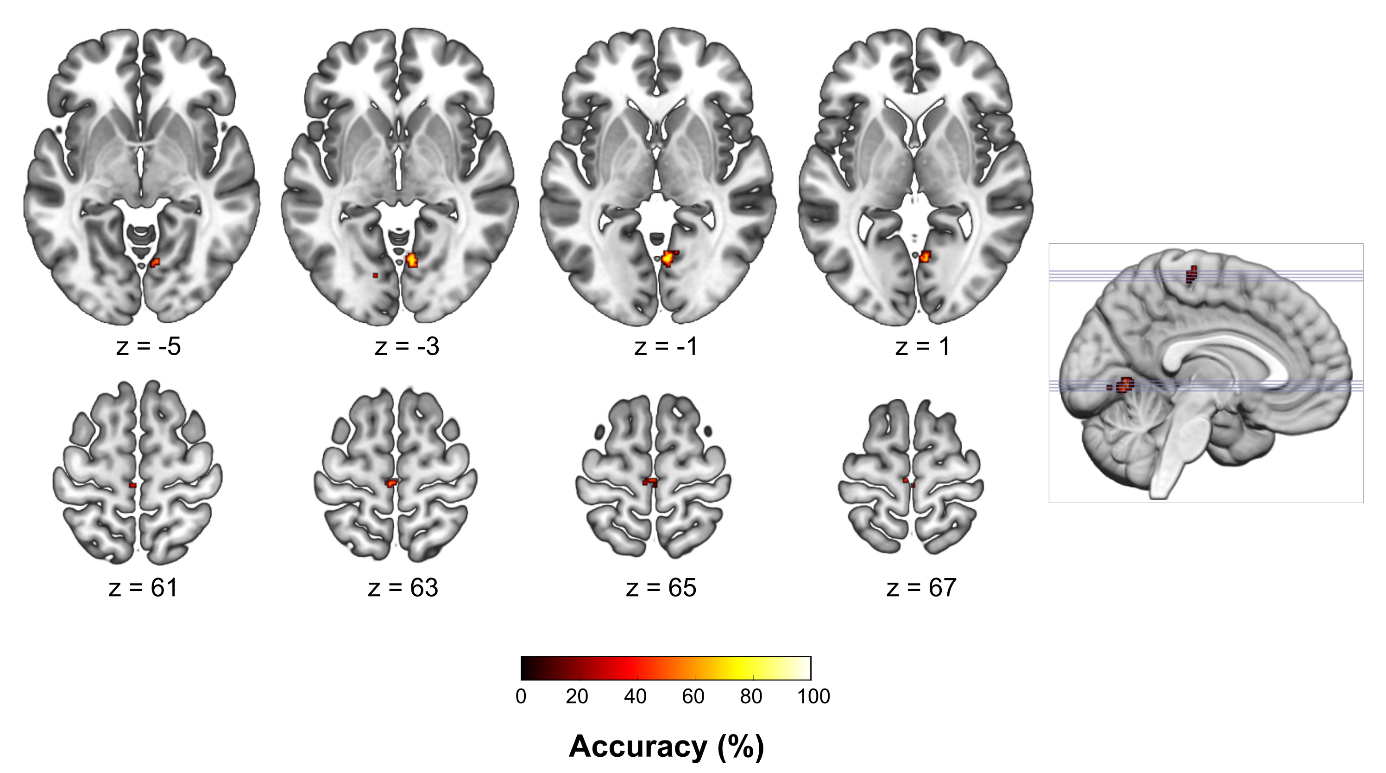
The significant clusters cover parts of the right lingual gyrus (n = 2 clusters of 52 and 1 voxel) and the left paracentral lobule (n = 2 clusters of 13 and 1 voxel). The classification accuracy under the threshold p < 0.05, FWE-corrected ranged from 75.5% to 78.6%.

**Table S12: Classification results of grey matter volume in regions-of-interest.**

| **ROI** | **MVPA Accuracy (%)** | **Specificity** | **Sensitivity** | **AUC** | **p-value** |
| --- | --- | --- | --- | --- | --- |
| ACC L | 73.96 | 54.76 | 83.15 | 0.72 | 0.0006** |
| ACC R | 67.14 | 26.19 | 86.52 | 0.54 | 0.06 |
| Amygdala L | 67.97 | 0 | 100 | 0.54 | 0.99 |
| Amygdala R | 67.2 | 0 | 98.88 | 0.61 | 0.99 |
| Cerebellum | 59.4 | 33.33 | 71.91 | 0.52 | 0.30 |
| Fusiform L | 51.21 | 23.81 | 64.04 | 0.46 | 0.94 |
| Fusiform R | 60.27 | 35.71 | 71.91 | 0.59 | 0.21 |
| Hippocampus L | 61.87 | 2.38 | 89.89 | 0.48 | 0.95 |
| Hippocampus R | 63.35 | 4.76 | 91.01 | 0.55 | 0.87 |
| IFG L | 51.98 | 26.19 | 64.04 | 0.49 | 0.85 |
| IFG R | 61.21 | 40.48 | 70.79 | 0.55 | 0.09 |
| MFG L | 62.64 | 40.48 | 73.03 | 0.63 | 0.04* |
| MFG R | 58.08 | 40.48 | 66.29 | 0.56 | 0.29 |
| SFG L | 61.76 | 47.62 | 68.54 | 0.62 | 0.08 |
| SFG R | 56.43 | 33.33 | 67.42 | 0.53 | 0.44 |
| OFC L | 67.97 | 45.24 | 78.65 | 0.68 | 0.0008** |
| OFC R | 64.12 | 42.85 | 74.16 | 0.62 | 0.03* |
| Insula L | 61.87 | 28.57 | 77.53 | 0.6 | 0.34 |
| Insula R | 68.68 | 40.48 | 82.02 | 0.66 | 0.01* |
| Putamen L | 73.41 | 40.48 | 88.76 | 0.72 | 0.002** |
| Putamen R | 68.08 | 30.95 | 85.39 | 0.63 | 0.21 |
| PHG L | 64.18 | 21.43 | 84.27 | 0.57 | 0.62 |
| PHG R | 67.2 | 40.48 | 79.77 | 0.72 | 0.16 |

Multivariate Pattern Classification Analysis (MVPA) results for the region-of-interest (ROI)-based grey matter volumes. The p-value indicates the significance of the original MVPA, and was obtained from non-parametric permutation testing using 5000 permutations. Abbreviations: ACC, anterior cingulate cortex; AUC, area under curve; Fusiform, fusiform gyrus; IFG, inferior frontal gyrus; MFG, middle frontal gyrus; OFC, orbitofrontal cortex; PHG, parahippocampal gyrus; SFG, superior frontal gyrus. * p < 0.05, uncorrected for multiple testing ** p < 0.05 Bonferroni corrected (p_Bonferroni_ < 0.0022).

**Table S13:** **Correlations between grey matter volume and DRSP scores in women with premenstrual dysphoric disorder (N=55).**

| **DRSP score** | **Direction** | **Cluster size** | **p_FWE_** | **MNI coordinates** | | | **AAL** |
| --- | --- | --- | --- | --- | --- | --- | --- |
|  |  |  |  | **x** | **y** | **z** |  |
| Total DRSP | N.S. | | | | | | |
| Irritability | N.S. | | | | | | |
| Depression | Negative | 81 | 0.055 | 2 | -59 | -9 | Vermis_4_5 |
| Affective lability | Negative | 42 | 0.087 | -32 | -48 | -41 | Cerebellum_8_L |
|  |  |  | 0.097 | -39 | -54 | -41 | Cerebellum_crus1_L |
| Anxiety | N.S. | | | | | | |

Voxel-wise partial correlations adjusted for TIV, age and BMI were conducted inside a mask combining the significant clusters resulting from the GMV group comparison between women with PMDD and controls. The total DRSP score, and the scores of the four core PMDD symptoms, namely irritability (items “irritable” and “conflicts”), depression (items “depressed”, “hopelessness” and “guilt”), affective lability (items “mood swings” and “easily hurt”) and anxiety (item “anxious”), were tested. Trend-level negative correlations were found between GMV in the cerebellum and the severity of depression and affective lability symptoms (p_FWE_<0.1). Abbreviations: AAL, Automatic Anatomical Labelling atlas; FWE, Family Wise Error correction; PMDD, premenstrual dysphoric disorder; L, left; N.S., non-significant.

**Figure S3: Correlations between grey matter volume and DRSP scores in women with premenstrual dysphoric disorder (N=55).** *Upper:* brain slices depicting the results of voxel-wise partial correlations adjusted for TIV, age and BMI conducted inside a mask combining the significant clusters resulting from the GMV group comparison between women with PMDD and controls. The DRSP scores for depression (items “depressed”, “hopelessness” and “guilt”), and affective lability (items “mood swings” and “easily hurt”) showed trend-level (p_FWE_<0.1) negative correlations with GMV in the cerebellum vermis and lobule VIII/crus 1, respectively. *Lower:* corresponding scatter plots illustrating the correlation between the mean GMV inside the clusters and the DRSP scores. Abbreviations: DRSP, Daily Record of Severity of Problems; FWE, Family Wise Error correction; GMV, grey matter volume; TFCE, Threshold-Free Cluster Enhancement.


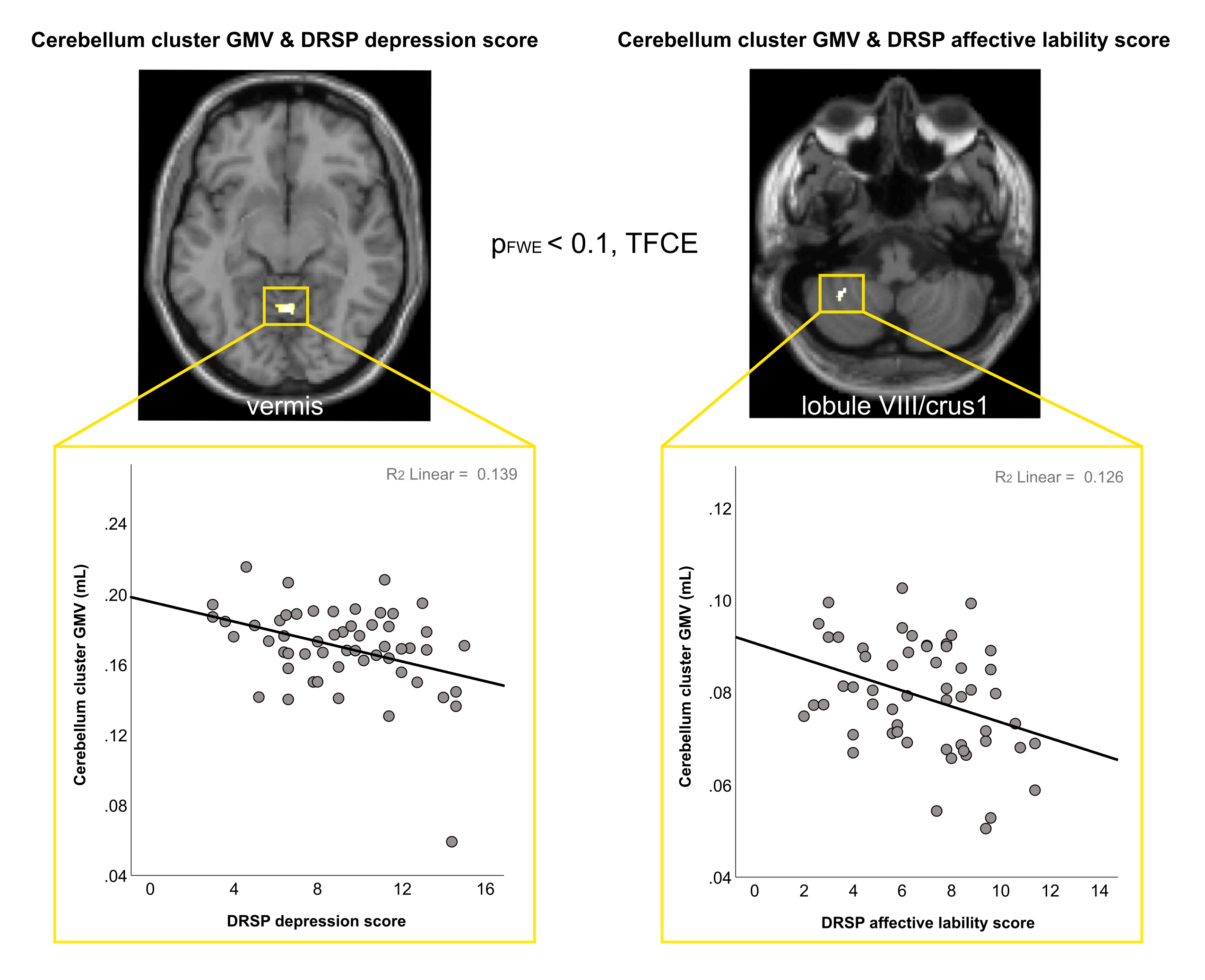

Supplement: Supplementary file 1 — supplement [file 41398_2022_2017_MOESM1_ESM.docx]
